# Supplementary material for: MetaRibo-Seq measures translation in microbiomes
Source: Nat Commun. 2020 Jun 29;11:3268. doi: 10.1038/s41467-020-17081-z (PMC7324362; doi:10.1038/s41467-020-17081-z)
Supplement: Supplementary file 10 — Supplementary Data 7 [file 41467_2020_17081_MOESM10_ESM.zip › File2/Confidence_VeryHigh_Taxonomy/368070_out.krona.html]

Javascript must be enabled to view this page.

members
magnitude
magnitudeUnassigned
count
unassigned
taxon
rank

368070\_out

6

5
2
superkingdom

phylum
5
1239

5
186801
class

order
5
186802

family
186803
1

genus
830
1


SRS012849\_contig\_number\_35858
species
1
45851

family
541000
4

4
1263
genus

46228
4
species

SRS015890\_contig\_number\_25412SRS050752\_contig\_number\_2838SRS074964\_contig\_number\_1223SRS148159\_contig\_number\_contig-100\_832.331606

1

SRS143895\_contig\_number\_22497
